# Prediction Models for Estimating Compressive Strength of Concrete Made of Manufactured Sand Using Gene Expression Programming Model

**DOI:** 10.3390/ma15175823

**Published:** 2022-08-24

**Authors:** Kaffayatullah Khan, Babatunde Abiodun Salami, Arshad Jamal, Muhammad Nasir Amin, Muhammad Usman, Majdi Adel Al-Faiad, Abdullah M. Abu-Arab, Mudassir Iqbal

**Affiliations:** 1 Department of Civil and Environmental Engineering, College of Engineering, King Faisal University, P.O. Box 380, Al-Hofuf 31982, Saudi Arabia; 2 Interdisciplinary Research Center for Construction and Building Materials, Research Institute, King Fahd University of Petroleum and Minerals, Dhahran 31261, Saudi Arabia; 3 Transportation and Traffic Engineering Department, College of Engineering, Imam Abdulrahman Bin Faisal University, P.O. Box 1982, Dammam 31451, Saudi Arabia; 4 Interdisciplinary Research Center for Hydrogen and Energy Storage (IRC-HES), King Fahd University of Petroleum & Minerals (KFUPM), Dhahran 31261, Saudi Arabia; 5 Department of Civil Engineering, University of Engineering and Technology, Peshawar 25120, Pakistan

**Keywords:** manufactured sand, concrete, compressive strength, gene expression programing

## Abstract

The depletion of natural resources of river sand and its availability issues as a construction material compelled the researchers to use manufactured sand. This study investigates the compressive strength of concrete made of manufactured sand as a partial replacement of normal sand. The prediction model, i.e., gene expression programming (GEP), was used to estimate the compressive strength of manufactured sand concrete (MSC). A database comprising 275 experimental results based on 11 input variables and 1 target variable was used to train and validate the developed models. For this purpose, the compressive strength of cement, tensile strength of cement, curing age, *D_max_* of crushed stone, stone powder content, fineness modulus of the sand, water-to-binder ratio, water-to-cement ratio, water content, sand ratio, and slump were taken as input variables. The investigation of a varying number of genetic characteristics, such as chromosomal number, head size, and gene number, resulted in the creation of 11 alternative models (M1-M11). The M5 model outperformed other created models for the training and testing stages, with values of (4.538, 3.216, 0.919) and (4.953, 3.348, 0.906), respectively, according to the results of the accuracy evaluation parameters root mean square error (RMSE), mean absolute error (MAE), and coefficient of determination (R^2^). The R^2^ and error indices values revealed that the experimental and projected findings are in extremely close agreement. The best model has 200 chromosomes, 8 head sizes, and 3 genes. The mathematical expression achieved from the GEP model revealed that six parameters, namely the compressive and tensile strength of cement, curing period, water–binder ratio, water–cement ratio, and stone powder content contributed effectively among the 11 input variables. The sensitivity analysis showed that water–cement ratio (46.22%), curing period (25.43%), and stone powder content (13.55%) were revealed as the most influential variables, in descending order. The sensitivity of the remaining variables was recorded as *w*/*b* (11.37%) > *f_ce_* (2.35%) > *f_ct_* (1.35%).

## 1. Introduction 

In a 2016 study published by Freedonia, approximately 52 billion metric tons of natural sand (NS) was used in 2019 for construction alone, making it the third most consumed material in the world after air and water [1]. This staggering piece of data revealed that the current unsustainable and non-renewable consumption of NS has led to severe reduction in the available resource, which sets the stage for a disproportionate increase in the value of materials [2]. For instance, the need for flood control in China, limiting mining, environmental protection policies, and a demand increase for sustainable construction have led to huge shortages in NS, making it very expensive [3]. In addition, the extreme exploitation of NS, especially the dredged river sand, has threatened the safety of bridges, stability of the river banks, and survival of the ecosystem [4,5], and there are some places without NS resources [6]. The properties of concrete are widely modified using a variety of aggregates and constituent materials [7,8,9,10]. There is need for a strategy to functionally and significantly replace the fast-depleting NS, with new material to mitigate the damage done to the ecosystem by draining its resources. According to studies, alternative sources for river sand include manufactured sand (MS), industrial by-products (certain types of slag, bottom ash), recycled aggregates, and so on.

MS from virgin rocks presents itself as a convenient, practical, sustainable, and economic substitute for NS in the production of concrete. Usually, in the manufacturing process of the sand, finer particles are unavoidably generated, where particles less than 75 µm are referred to as stone dust or powder. The standards governing the recommended content of stone dust or powder vary however, a maximum of 7% and 10% of dust or powder content are the recommendation for ASTM C33 [11] and Chinese [12] standards, respectively. Due to the manufacturing process of breaking and bringing, there are differences in the particle shapes between the NS and the manufactured sand. Generally, the sand grains of the manufactured sand show distinctive rough-angular particles, which have the ability to yield granular critical state frictional angles [13,14,15]. As a result of improved interlocking between the rough-angular sand particles, there is a positive influence on the mechanical strength and durability properties of concrete [16]. In addition, better bonding properties were reportedly [17] achieved with reinforced concrete also prepared with manufactured sand. Many works [18,19,20] have been conducted to study the comparative effect of manufactured sand and NS on the compressive strength of concrete. They have largely reported the superior performance of the concrete prepared with manufactured sand over NS. In the study by Li et al. [21], concrete was developed using manufactured sand processed from different sandstones, such as limestone, quartzite, granite, basalt, and granite gneiss. It was mentioned that morphology and texture characteristics of the manufactured sand greatly affected the performance of the concrete. One of the properties used in evaluating its performance is compressive strength. 

Compressive strength remains one of the most important parameters to characterize concrete generally [22] and, specifically, manufactured-sand concrete (MSC). The process involved in experimentally obtaining the compressive strength of concrete in the laboratory is intricate and cost- and time-consuming with a limited testing period. Despite all the time spent in the laboratory, it was practically impossible to adequately explore all the different combinations of the mixture compositions (i.e., cement, coarse aggregate, water, manufactured sand, etc.). Machine learning (ML)–based techniques were recently and successfully deployed to predict the compressive strength of concrete [23,24]. Ly et al. [25] proposed principal component analysis (PCA) with teaching–learning-based optimization (TLBO) as enhancement to the prediction accuracy of adaptive neuro-fuzzy inference system (ANFIS) in predicting the compressive strength of concrete prepared with manufactured sand. Similarly, hybrid artificial intelligence (AI) of particle swarm optimization (PSO)–based adaptive network-based fuzzy inference system (PSOANFIS) and a genetic algorithm (GA)–based adaptive network-based fuzzy inference system (GAANFIS) was proposed by Dao et al. [26] to predict the 28-day compressive strength of GPC containing 100% waste slag aggregates. In a recent study, Feng et al. [27] proposed an intelligent approach that employs an adaptive boosting (AdaBoost) algorithm to predict the compressive strength of concrete. The superior predictive ability of AdaBoost was proven by comparing its performance with those of artificial neural networks (ANN) and support vector regression (SVR). In another attempt, Duan et al. [28] proposed an ANN model for investigating the compressive strength of concrete prepared with varying types and sources of recycled aggregates. Generally, from the studies of the proposed models [29,30,31], it was clear and proven that ML has the capabilities of modeling the nonlinearity inherent between the concrete mixtures to better predict the compressive strength of concrete. In a recent study by Ren et al. [32], ensemble classification and regression tree (En_CART) techniques were employed in predicting the compressive strength of the manufactured sand concrete. Comparatively, the predictive performance of ANN, Gaussian process regression (GPR), RF, and SVR were also studied with En_CART models was established in predicting the compressive strength of the developed manufactured sand concrete with 1350 target variables from 328 concrete mixture designs. Saridemir [33] explored gene expression programming (GEP) to develop a model splitting the tensile strength from the compressive strength of concrete. In building the GEP-based model, 536 experimental datasets from available literature were used to develop a formulation for splitting the tensile strength of concrete as a function of specimen age and cylinder compressive strength. A separate 173 experimental datasets were used to validate the formulations other than the training and testing data. The obtained results from the GEP-based model were compared with experimental results, the regression-based models and national building codes formulas and were found to agree well with the experimental data. In other works, GEP has been successfully utilized to predict the compressive strength of concrete [34,35,36]. 

In this study, the goal is to develop a GEP-tree-based prediction model with strong nonlinear capabilities for better estimation of the compressive strength of concrete developed with MS and reveal the relationship between the features. The datasets were pulled from the works of Zhao et al. [37] with included input parameters, such as mixture compositions, water content, cement content, manufactured sand properties, and curing days. The target response from the developed ML model is the compressive strength of concrete with manufactured sand. For model evaluation, statistical parameters, such as root mean square error (RMSE), mean absolute error (MAE), coefficient of determination (R^2^), and coefficient of correlation (R) were used. The rest of the paper is laid out as follows: The Section 2 contains information on the datasets and the GEP-tree model that was used to train them. The outcomes of the investigation are described in the Section 3. Finally, the study’s principal conclusions are provided. The suggested model will be used to choose the best mixture design for MSC in order to attain the desired compressive strength as specified by applications.

## 2. Methodology

This section describes the experimental database used for the development of AI models. Additionally, GEP modeling and evaluation criteria are also presented. 

### 2.1. Experimental Database

**Figure 1:**
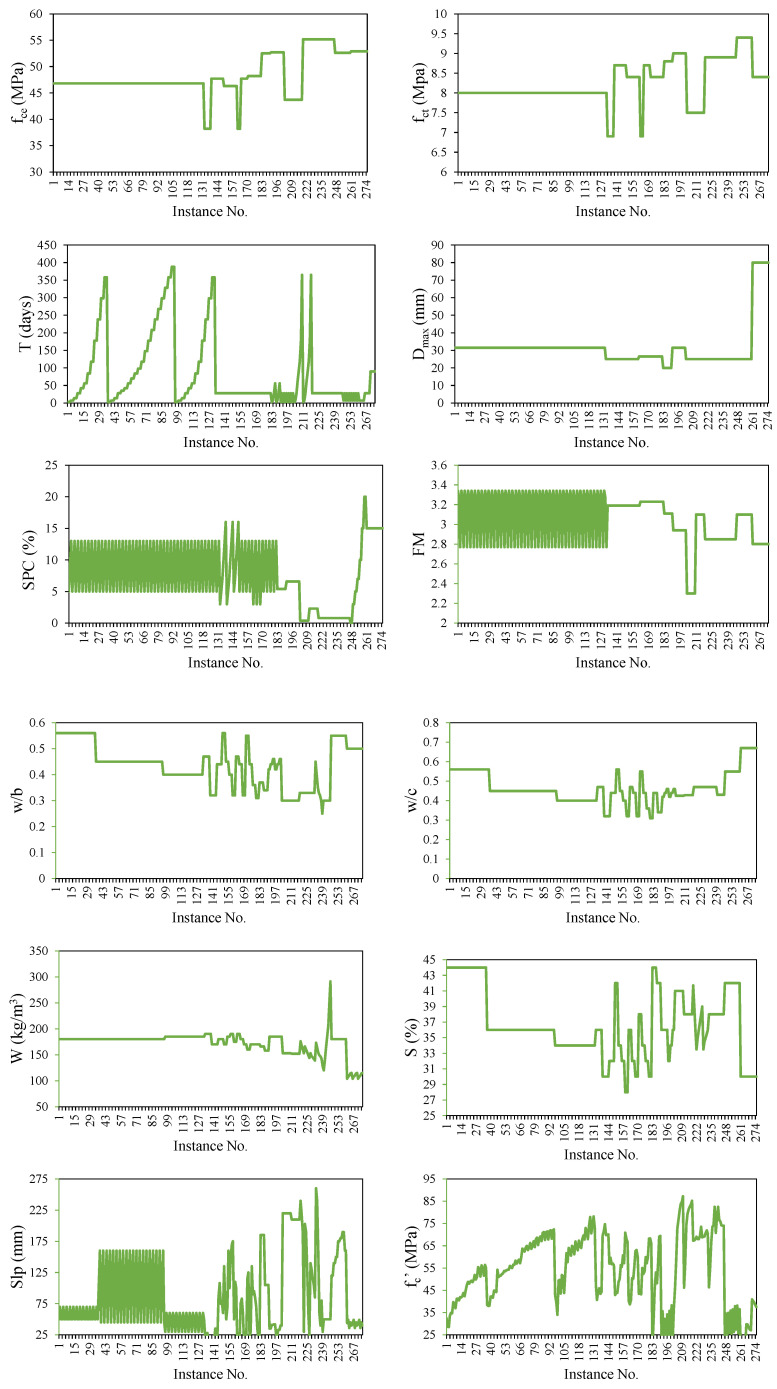
Details of variables used in the development of models.

It is critical to establish a well-assembled and vast dataset with clear and explicit descriptions, insights, and statistically significant input variables if the goal is to generate powerful and resilient ML models. As a result, the GEP-tree-based algorithms used in this study were trained using a cleaned database, including 275 experimental datasets with 11 features taken from the data-in-brief [37,38] worked by Zhao et al. [39]. The models were developed for the compressive strength (*f_c_’*) of MSC using eleven recorded attributes as inputs: compressive strength of cement (*f_ce_*, MPa), tensile strength of cement (*f_ct_*, MPa), curing age (*T*, days), *D_max_* of crushed stone (mm), stone powder content (*SPC*, %), fineness modulus of sand (*FM*), water-to-binder ratio (*w/b*), water-to-cement ratio (*w/c*), water (*W*, kg/m^3^), sand ratio (*S*, %), and slump (*Slp*, mm). The proper amount of stone powder in manufactured sand helps in improving the workability of MSC [40]. This is a result of the paste’s thickening consistency brought on by the higher water absorption of the stone powder and the larger volume of paste that contains the pulverized stone powder. In the experimental study by Zhao et al. [39], limestone-based manufactured sand with a particle size of around 0–4.75 mm, continuous-graded crushed limestone in the range of 5 to 31.5 mm, and grade 42.5 ordinary silicate cement was employed in the MSC. Stone powder was classified as having a particle size less than 0.075 mm, and its mass in manufactured sand was modified to 5 percent, 9 percent, and 13 percent. All mixtures also included tap water and the high-performance water reducer FDN-1, which has a water reducing rate of 19%. The water absorbed by the stone powder was included in the initial mixing water, the sand ratio was raised by around 2% compared to concrete made with natural sand, and decreased by 1–2% for every 3% increase in stone powder by mass when using manufactured sand. In addition to cement, other binders, namely fly ash and silica, were also used; therefore, the input attribute was named the water-to-binder ratio. Table 1 summarizes the statistical evaluation and their individual data of the datasets (input and target parameters) used for model development. The distribution charts of the input and target variables utilized during model training, in terms of their magnitudes along observation numbers, are shown in Figure 1. Plotting these numbers may aid in identifying parameters for which there are insufficient data, and more data are needed. Because the input parameters are interdependent, all variables studied were correlated, and the findings are displayed in Table 2. More than half of the input variables are positively associated, according to a quick evaluation of their effect on the target variable.

### 2.2. GEP Modeling 

**Figure 2:**
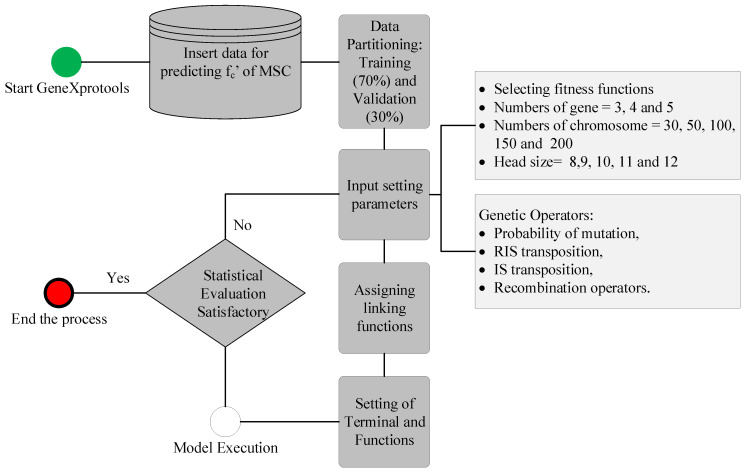
Flowchart of the study.

Gene expression programming (GEP) was first proposed by Koza in 1992 and was inspired by Darwin’s idea of natural selection and evolution. GEP has been successfully employed for tackling numerous complicated engineering challenges due to a number of its inherent advantages [41]. The GEP algorithm has been successfully used for a variety of concrete-related applications [42,43,44,45,46,47]. GEP uses a population-based technique, which is inspired by the traditional genetic algorithms (GAs) procedure for prediction and optimal solution finding. The procedure starts with initialization, which entails creating a random population, and then moves on to producing differences in the parent population by genetic operators such as crossover, elitism, and mutation. The fitness values of the population passed to the following generation are used to make decisions. Figure 2 depicts the flowchart for a common GEP model’s essential functioning mechanism. All of the stages are applied in order to diversity and enrich the offspring population. The number of genes, chromosomes, head size, genetic operators (mutation and crossover), maximum number of generations, and connecting functions all affect the predictive effectiveness of the GEP model. 

The GEP model was chosen for the study because it provides a simple and interpretable mathematical prediction model that can be utilized with high confidence by researchers and practitioners in the field for similar situations without the requirement for lengthy experimental testing. 

GeneXprotools v5.0 created by Candida Ferreira (Portugal) was used to construct the GEP models. The data was first retrieved into the tool interface, where the attributes were separated into target (output) and input (explanatory) variables. The data were divided into training and validation groups at random. Previous research has shown that partitioning in the 70/30 ratio produces the best results [48]. The same partitioning percentages were used in the current investigation. The next stage involved adjusting the setting parameters of the model. Additionally, probability of mutation and crossover technique was used as genetic variation. In this regard, the number of chromosomes was varied between 30 and 200, and head sizes were varied between 8 and 12). Because of the intricacy of the output’s mathematical equation, the number of genes has a significant impact on the model’s performance. Previously, majority of researchers employed different of genes, i.e., 3 [49], 4 [42], and 5 [47]. Increasing the number of genes may boost performance, but it will also complicate the output’s mathematical equation, and thus the number of genes in this analysis ranged from 3 to 5. Different linking functions between the genes were investigated; however, addition produced the best results, and hence it was used in this study. The fitness function used in this mode was RMSE. The trial details are shown in the diagram below. 

### 2.3. Evaluation Criteria

Model evaluation was done using various statistical indices, such as the correlation coefficient (R), root mean square error (RMSE), and mean absolute error (MAE), obtained from the previous research [49]. The R value is a number that varies from 0 to 1, with 1 indicating perfect correlation and values approaching zero indicating a very poor connection between the predictors and the target variable. R values of 0.8 and above have been widely accepted as yielding a more robust and accurate prediction of projected values.

## 3. Results and Discussion

This section presents performance evaluation of the proposed GEP models. Analysis of selecting the best hyperparameter settings for the GEP model is also discussed. The model’s performance is analyzed using different statistical indices, regression slopes, and the predicted-to-experimental ratio. GEP formulations obtained from the best fit model are also presented. Finally, results of a detailed parametric analysis (showing the relative influence of predictors on the target variable) are presented. 

### 3.1. Effect of Genetic Variables 

**Figure 3:**
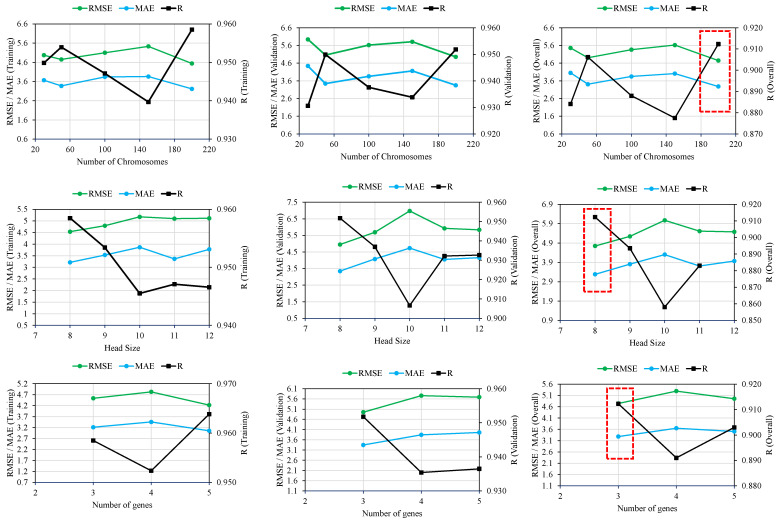
Effect of genetic variables on the performance of GEP models.

**Figure 4:**
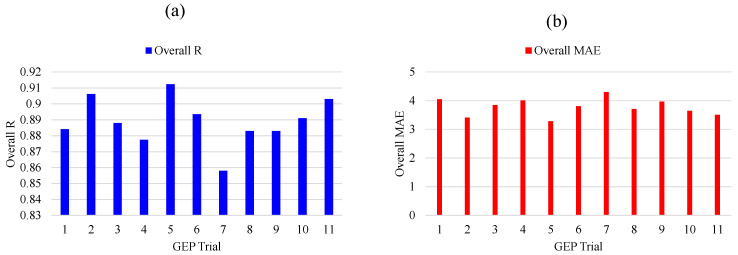
Comparison of overall (**a**) R and (**b**) MAE for the trials undertaken in this study.

A total of 11 trials (M1 to M11) were run to discover the best hyperparameter values for the problem. The number of chromosomes, head sizes, and number of genes were varied under several combinations/permutations, as shown in Table 3 and Figure 3. Initially, chromosome sizes were varied from 30 to 200 while keeping the head size at 8 and the number of genes at 3. The results indicated that a chromosome size of 200 achieved the optimal model performance. The head size was then adjusted from 8 to 12 while keeping the chromosomes (200) and genes (3) at their fixed values. The highest model performance was observed when the head size was kept at 8. Finally, a number of genes were changed using the above optimum values for the number of chromosomes (200) and head size (8). The best model performance in this case was attained by fixing the number of genes at 3. To summarize, the proposed model performed better when the number of chromosomes, head size, and number of genes were set to 200, 8, and 3, respectively. Figure 3 presents better visual illustration of model performance evaluation based on the considered metrics (R, RMSE, MAE) as the genetic parameters were adjusted in successive steps. Figure 4 shows the predictive performance of the 11 GEP models based on overall MAE and R values. It may be noted from the Figure 4 that M5 exhibits the highest R values and lowest R value, showing its robust and superior performance compared to other models. Thus, the succeeding analyses were based on the M5 model setting. 

### 3.2. Performance of the Developed Models

This section describes the predictive performance of the developed GEP models based on statistical evaluation indices, slope of regression line, and ratio of predicted/experimental (pred./exp) ratios. For developing the model, the ratio between the number of experimental records (i.e., 70% training and 30% validation data points), which in this case were 192 and 83, were considered. Models were developed based on the 11 recorded attributes mentioned in Section 2.1. The previous literature in this regard suggests that the ratio of data points to number of input predictors must not be less than three and should preferably exceed five for the development of an efficient predictive model [50]. This ratio is significantly above the required limit for the considered compressive strength estimation of concrete manufactured with sand (17.45 in the training set and 7.54 in the validation set) in this study, indicating a more reliable ML model.

#### 3.2.1. Statistical Evaluation

**Figure 5:**
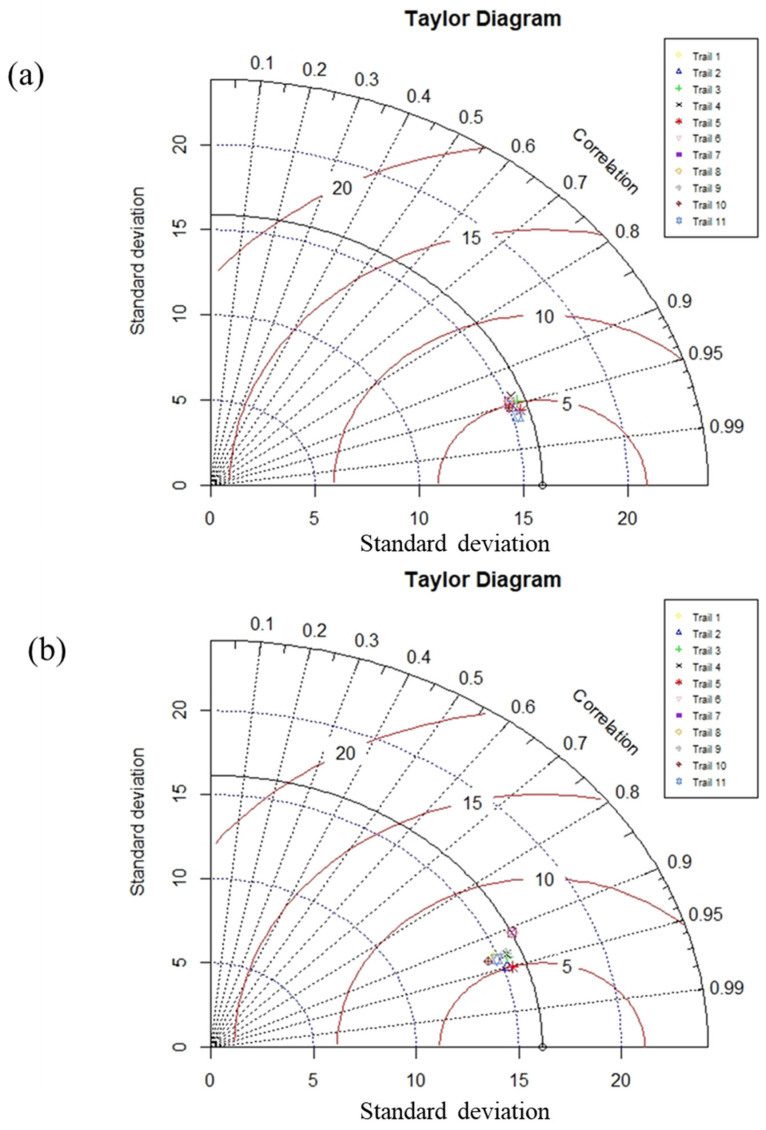
Comparison of the trials using Taylor diagram for (**a**) training data and (**b**) validation data.

Table 3 shows the statistical evaluation of experimental (actual) and prediction results of the GEP model for the compressive strength (CS) of concrete (manufactured with sand). The results are shown for both the training and validation stages. The overall values of R in the GEP predictive model are in general higher than 0.88, indicating that the experimental and projected outcomes are in close agreement. Considering the R^2^ values, it is apparent that M5 achieved the highest value (0.912), outperforming other models. The experimental results show that R^2^ values for M5 models in both the training and validation sets are comparable and are also higher than other models. However, it is widely acknowledged that merely a greater R^2^ is not a unique or reliable measure of the robustness and superiority of an AI model [51]. Consequently, other key indices such as MAE and RMSE were used for the current study for better comparative analysis of the selected GEP models. It may be observed from the results (Table 3) that the achieved RSME (4.953) and MAE (3.348) confirm the superiority of the M5 model and its improved prediction performance. Based on the considered statistical indices, M2 is identified as the next best model. The CS prediction results in Table 3 suggest that all of the formulated GEP models yielded satisfactory prediction performance. The robustness of all formulated models is evident from the Taylor diagram shown in Figure 5. Factors such as the GEP model’s algorithmic structure, diverse reproduction process, stochastic adaptive genetic operators, and minimum assumptions about the input data structure [51] are responsible for such precise and accurate predications achieved by GEP model. Furthermore, the GEP algorithm generates random choices and functions that are in line with earlier experimental findings [48]. The GEP model has comparable performance to previously developed AI models such as random tree, multilinear regression, M5P, stochastic M5P, random forest, Gaussian process, and bagged M5P tree; however, it outperforms other AI models in terms of yielding a simple mathematical equation, whereas previously developed models are mainly criticized for their black-box processing of input information. In order to estimate the uniaxial CS of manufactured-sand concrete, Zhao et al. [52] studied two ANN-based scenarios. First, nine regular algorithms were used to train the ANN, and the best one was chosen to represent the traditional ANN (CNN). In the second scenario, two enhanced ANNs using the biogeography-based optimization (BBO) and multi-tracker optimization techniques were produced (MTOA). The CNN’s performance in comparison to hybrid models revealed that BBO and MTOA can both build an ANN that is more accurate. The most accurate model yielded MAE of 3.8529 and 3. 8759 for the training and testing data, respectively. This study developed a model having MAE of 3.216 and 3.348 for the training and validation data, showing superior performance compared with the previously developed model. Nevertheless, the current model was translated in the form of a simple mathematical equation. 

#### 3.2.2. Comparison of Regression Slopes and Error Analysis

**Figure 6:**
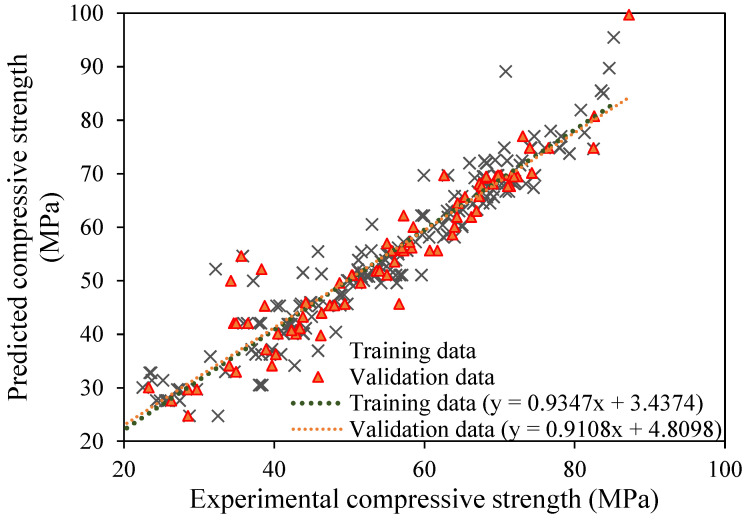
Comparison of regression slope for trial No. 5.

**Figure 7:**
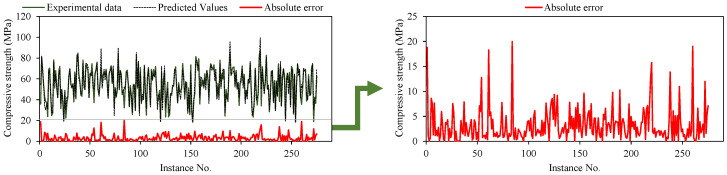
Error Analysis for the optimized trial.

Another commonly used evaluation metric for assessing the suitability of AI/statistical models is the slope of the regression lines, which implies the trend between actual (experimental) and predicted output values [44]. The research analysis also evaluated the performance of developed GEP models based on regression slopes, and the results for the best-fit model (M5) are shown in Figure 6. The trend lines (regression slope) for both training and validation data are shown. Further, the corresponding predictive equations for training and validation stages are also given in the same figure. A standard 45-degree crossing through the diagonal represents the optimum fitting line which has a slope of unity (1). The error indices, such as RMSE and MAE, have minimal values for a regression line with a slope approaching 1 and correlation values of 0.8 and above [53,54]. Closer distribution of the depicted points with reference to the standard diagonal line are indicative of more acceptable and reliable model performance. As shown in Figure 6, the slope value of the regression line for training data is 0.93 and that for validating stage data is 0.91, implying excellent prediction performance of M5. The shown regression lines for the selected M5 model show that, in general, plotted points are concentrated around the trend line, indicating that it performs reasonably well. It should be observed that the regression slope values for the validation data are comparatively lower compared to the corresponding values for the training stage data, indicating that no overfitting problem exists. Figure 7 presents the error analysis plot for the optimized trial (M5) against the number of observed instances. The absolute error (difference between predicted and actual values) is consistently lower than 10 percent for the majority of instances, suggesting that a lower disparity exists between the experimental and predicted results. 

#### 3.2.3. Predicted-to-Experimental Ratio

**Figure 8:**
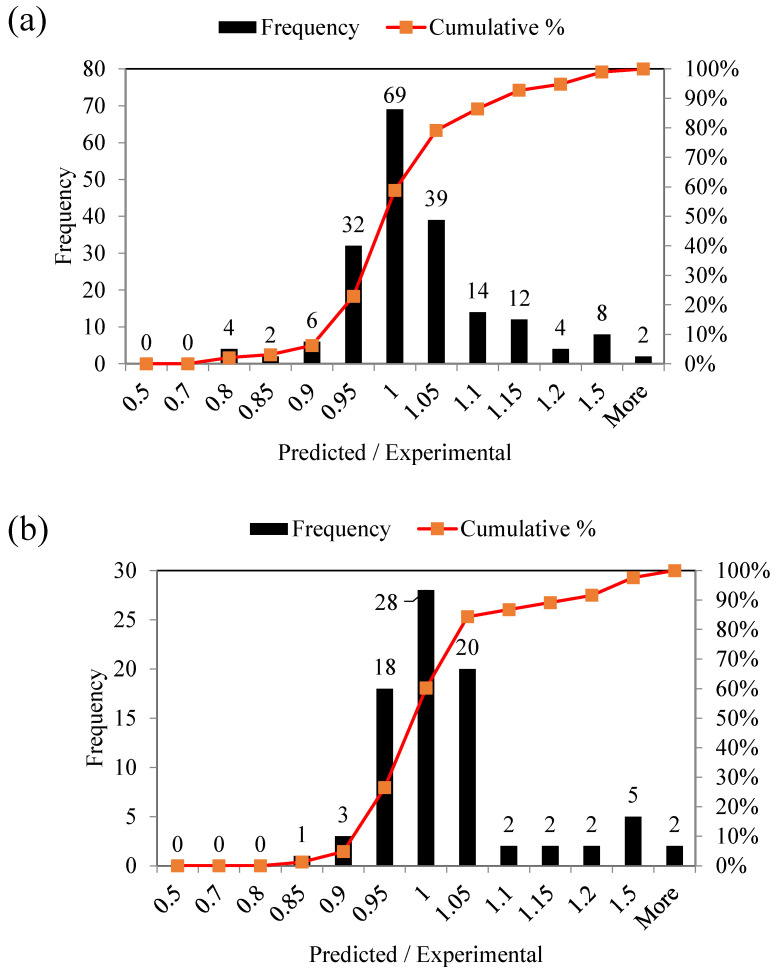
Predicted/experimental ratio for the optimized trial No. 5. (**a**) Training dataset (**b**) Validation dataset.

The frequency ratio and cumulative percentage of the model’s predicted results divided by the experimental data are illustrated in Figure 8. The results are shown for the ratio (predicted/experimental) between 0.5 and 1.5, showing a 50% deviation of predicted results from the experimental data. Pred/exp ratio results are shown for both training (Figure 8a) and validation (Figure 8b) datasets. The maximum number of observations fall between 0.950 to 1.10, indicating that the majority of data points are within 10% uncertainty. This shows that the forecasts produced by the models are reliable and accurate. Model M5 attained the greatest cumulative percent values of 83% ((6 + 32 + 69 + 39 + 14)/(192)) for training stages and 85% ((3 + 18 + 28 + 20 + 2)/(83)) for validation data within the bin range of 0.9 to 1.10. All of the developed models gave the maximum cumulative frequency within this bin range; however, model M5 had the largest percentage of both cumulative percentage and frequency ratio of pred/exp outputs. 

#### 3.2.4. GEP Formulations

**Figure 9:**
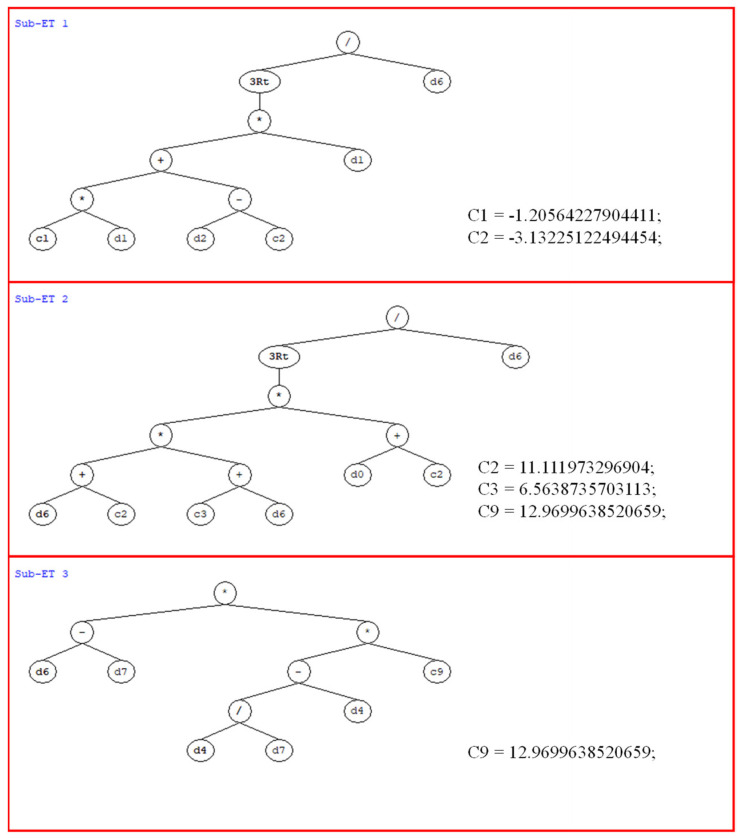
Expression tree generated from GEP model for optimized trial.

The ideal/optimum combination of GEP model parameters giving (M5) was employed in accordance with previous studies [43,48,55,56] for generating an empirical formulation to forecast the CS of concrete and expression tree shown in Figure 9. Equation (1) to Equation (4) illustrate the final empirical equation, which was produced by integrating several mathematical models obtained from the Matlab-based GEP model. The developed mathematical formulations shown in the equations below can be used for estimating the CS of concrete using the input variables $f_{ct}$, $f_{ce}$, w/b, w/c, *SPC*, and *T.* It is worth noting that the established model can be utilized to forecast the CS of concrete under typical circumstances with information on similar variables without the need comprehensive laboratory-based testing.

(1) $${f_{c}}^{\prime }=x+y+z$$

(2) $$x=\frac{\sqrt[{3}]{\left(\left(\left(-1.21\times f_{ct}\right)+\left(T+3.13\right)\right)\times f_{ct}\right)}}{w/b}$$

(3) $$y=\frac{\left(\left(\left(w/b\right)+11.11\right)\times \left(6.56\times \left(w/b\right)\right)\right)\times \left(f_{ce}+11.11\right)))\;}{w/b}$$

(4) $$z=((w/b-w/c)\times (\left(\frac{SPC}{w/c}\right)\times 12.97))$$

where $f_{ct}$ is the tensile strength of cement; $f_{ce}$ is the compressive strength of cement; w/b is the water-to-binder ratio; w/c is the water-to-cement ratio; *SPC* is the stone powder content; and *T* is the curing period. 

### 3.3. Sensitivity and Parametric Analysis

It is frequently important to test the efficacy of machine learning–based simulation on simulated datasets in order to establish and verify their validity on a variety of datasets. Sensitivity analysis and parametric analysis are two such techniques commonly used in this perspective that attempt to examine the effectiveness of selected GEP model predictions using the inter-dependence of physical phenomena [57,58,59,60]. Sensitivity analysis is frequently used for exploring the variation in response of the proposed predictive model with reference to any changes in the specific input variables/features [59,61,62], while parametric analysis is used to establish the relative importance of the predictors in predicting the target variable (CS in this case). For the current investigation, several predictors were subjected to a parametric analysis to determine their respective importance in relation to the CS of concrete. It is worth noting that all six variables used for both sensitivity analysis and parametric analysis were numeric, and hence the corresponding fluctuation on the target variable was easy to interpret. 

**Figure 10:**
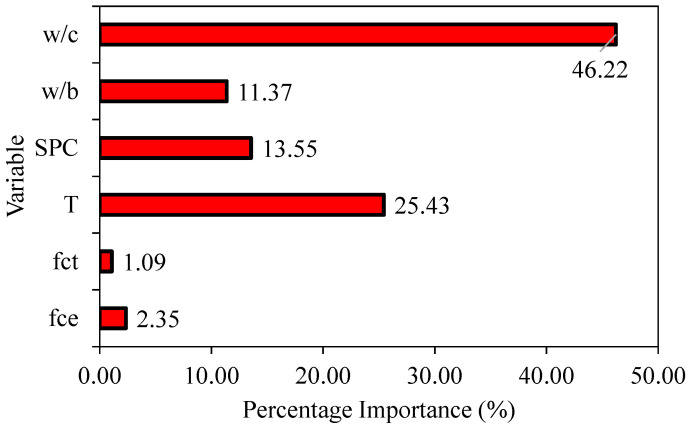
Sensitivity analysis of the developed model.

**Figure 11:**
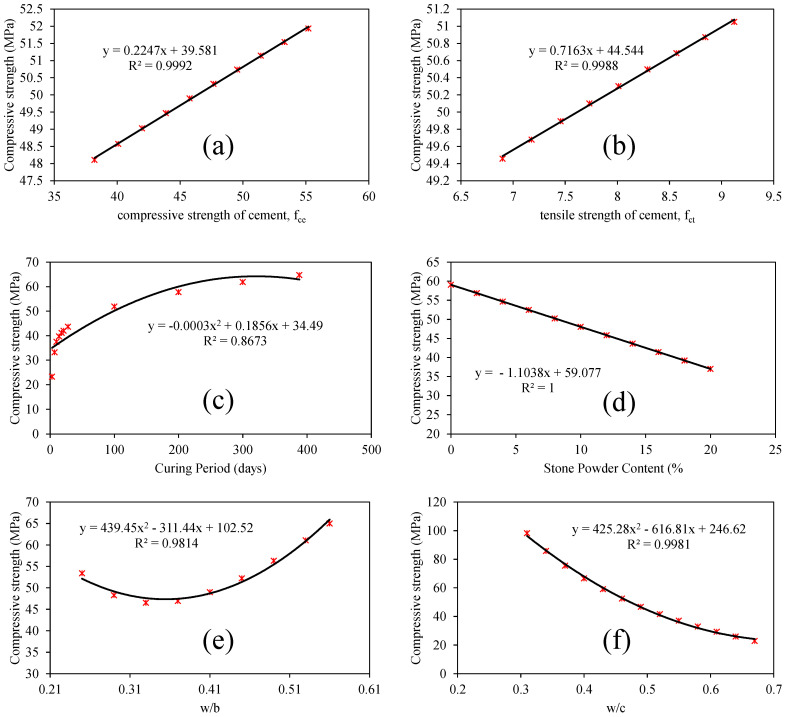
Parametric analysis of the optimized model (**a**) *f_ce_* (**b**) *f_ct_* (**c**) *T* (**d**) *SPC* (**e**) *w*/*b* (**f**) *w*/*c*.

Figure 10 shows the sensitivity analysis for the investigated predictors (tensile strength of cement; compressive strength of cement; water-to-binder ratio; water-to-cement ratio; stone powder content; and curing period). It may be noted that the variables water-to-cement (*w*/*c*) ratio, curing period (*T*), and stone powder content (*SPC*) are highly sensitive, implying any variations in these variables will strongly dictate the prediction performance of developed GEP models. On the other hand, predictors such as tensile and compressive strength of concrete were identified as comparatively less sensitive. Figure 11a–f illustrates the detailed parametric analysis of the optimized model (M5) considering the same predictors. The trend line/slope and the corresponding R^2^ values for each are also shown in Figure 11. As demonstrated in Figure 11a, the compressive strength of concrete (CS) is linearly associated with any increase in the tensile strength of the cement used. A similar trend for variable *f_ct_* (compressive strength of cement) is also observed (Figure 11b). The curve depicting the relationship between the CS and curing period (*T*) is also positively sloped; however, the trend is not perfectly linear (Figure 11c). There is a comparatively rapid increase in the CS during the first 200 days (6 months), and afterwards the trend continues to increase until it becomes steady/flat at *T* values of 250 and above. The CS of concrete and stone powder content (*SPC*) are inverse, and this is also reflected by the negatively sloped trend line plotted between the two (Figure 11d). Considering the influence of the water-to-binder (*w*/*b*) ratio on the CS of concrete (target), it may be observed that the initial increase in *w*/*b* values (until *w*/*c* approaches 0.36) is accompanied with a reduction in CS (Figure 11e), followed by rapid increase later for any further increase in *w*/*b* ratio. Finally, the impact of increasing *w*/*c* ratios on the CS is shown in Figure 11f, and it is revealed that increasing *w*/*c* values (from 0.35 to 0.68) are expected to lower the CS significantly. The above observations are consistent with a number of earlier investigations [63,64]. 

## 4. Conclusions

The excessive application of river sand, especially as a construction material, has led to severe shortage of natural resources and unbalanced river eco-system. This study investigates the nonlinear capabilities of the GEP prediction model for the compressive strength of concrete made of manufactured sand from stone powder as a partial replacement for normal sand. The following findings may be taken from this investigation:

1. The optimum statistical indices were acquired after 11 trials based on variable genetic parameters. These values for the training and validation datasets in the case of the ultimately selected model (trial 5) were RMSE (4.538 and 4.953) MPa, MAE (3.216 and 3.348) MPa, and R^2^ (0.919 and 0.906), respectively. Furthermore, the MAE values of the selected models show a mean error of 5.93 percent (training) and 6.17 percent (validation). These values are substantially lower, suggesting that the defined GEP models for forecasting compressive strength of MSC are reliable for use in future.
2. The Taylor diagram shows the robustness of all the models; however, it reveals the superiority of trial 5. Other statistical performance metrics, such as predicted-to-experimental ratio for the optimum trial and the slope of the regression line between experimental and anticipated outcomes, were employed to supplement the accuracy analysis of the best GEP model. The best model yielded 0.9347 (training) and 0.9108 (validation) regression slopes, which are closer to unity (i.e., ideal slope), reflecting the reliability of the developed model. The predicted/experimental ratio manifested that 85 percent and 83 percent of the values were within 10% of deviation from the actual experimental results.
3. The MATLAB code extracted from the final GEP model was used to create a mathematical equation with easily determinable input parameters to evaluate the compressive strength of MSC, obviating the need for time-consuming and expensive sample testing and thus affecting the cost-effectiveness of civil engineering projects. It was also determined that water–cement ratio, water–binder ratio, compressive strength of cement, tensile strength of cement, curing period and stone powder percentage are the six variables among the eleven effectively contributing to compressive strength.
4. The sensitivity analysis showed that the water-to-cement ratio is the most influential parameter followed by duration and percentage replacement of stone powder content, equaling 46.22, 25.43, and 13.55, respectively, in contributing to the compressive strength. The parametric analysis revealed that the compressive strength of concrete linearly changes with the tensile and compressive strength of cement. The increase in compressive strength of MSC was steeper during the first 100 days, which also validates the model in terms of its coherence with the literature. The increase in the percentage of stone powder decreased the compressive strength of the MSC. Maximum magnitude of compressive strength was obtained at a water–cement ratio of 0.30.
5. The model was based on the available literature, which covers specific ranges of the input variables. More robust models can be developed based on the literature from multiple sources covering a wider range.

## Figures and Tables

**Table 1:** Descriptive statistics of the input data used for the development of models.

Descriptive Statistics	*f_ce_* (MPa)	*f_ct_* (MPa)	*T* (Day)	*D*_max_ (mm)	*SPC* (%)	FM	*w*/*b*	*w*/*c*	W (kg/m^3^)	S (%)	Slp (mm)	Compressive Strength *f_c__’_* (MPa)
Mean	48.34	8.26	82.11	31.37	7.79	3.06	0.43	0.47	172.68	36.74	87.79	54.24
Standard Error	0.23	0.03	6.18	0.73	0.28	0.02	0.01	0.00	1.26	0.26	3.65	0.96
Median	46.80	8.00	28.00	31.50	7.00	3.19	0.45	0.45	180.00	36.00	60.00	55.40
Mode	46.80	8.00	28.00	31.50	13.00	3.34	0.45	0.45	180.00	36.00	50.00	68.00
Standard Deviation	3.77	0.53	102.49	12.16	4.64	0.25	0.08	0.08	20.96	4.33	60.60	16.00
Sample Variance	14.20	0.28	10,504.05	147.95	21.53	0.06	0.01	0.01	439.22	18.73	3671.81	256.00
Kurtosis	0.36	0.23	1.54	11.31	−0.94	0.24	−0.93	0.53	6.41	−0.75	−0.37	−0.72
Skewness	0.11	0.07	1.66	3.45	0.10	−0.84	−0.06	0.68	−0.81	0.28	0.89	−0.27
Range	17.00	2.50	385.00	60.00	20.00	1.04	0.31	0.36	187.00	16.00	249.00	68.80
Minimum	38.20	6.90	3.00	20.00	0.00	2.30	0.25	0.31	104.00	28.00	11.00	18.40
Maximum	55.20	9.40	388.00	80.00	20.00	3.34	0.56	0.67	291.00	44.00	260.00	87.20
Count	275	275	275	275	275	275	275	275	275	275	275	275.00
Confidence Level (95.0%)	0.45	0.06	12.17	1.44	0.55	0.03	0.01	0.01	2.49	0.51	7.19	1.90

**Table 2:** Pearson’s correlation coefficient among the variables used in the development of models.

	*f_ce_*	*f_ct_*	*T*	*D_max_*	*SPC*	*FM*	*w/b*	*w/c*	*W*	*S*	*slp*	*CS*
*f_ce_*	1											
*f_ct_*	0.88	1										
*T*	−0.26	−0.33	1									
*D_max_*	0.20	−0.04	0.01	1								
*SPC*	−0.17	−0.12	0.08	0.45	1							
*FM*	−0.25	−0.07	0.06	−0.20	−0.08	1						
*w/b*	−0.13	−0.05	0.10	0.30	0.46	0.23	1					
*w/c*	0.22	0.06	−0.02	0.63	0.27	−0.15	0.74	1				
*W*	−0.34	−0.12	0.13	−0.61	−0.04	0.35	0.12	−0.41	1			
*S*	−0.03	−0.03	0.09	−0.37	−0.22	−0.04	0.42	0.36	0.21	1		
*slp*	0.06	0.08	0.02	−0.27	−0.37	−0.06	−0.19	−0.03	−0.10	0.29	1	
*CS*	−0.14	−0.20	0.46	−0.40	−0.36	−0.06	−0.66	−0.59	0.09	−0.16	0.16	1

**Table 3:** Details of trials and their statistical evaluation using correlation and error indices.

Trial No.	Used Variables	No. of Chromosomes	Head Size	Number of Genes	Constants per Gene	No. of Literals	Program Size	Training Dataset				Validation Dataset				Overall R^2^
								Best Fitness	RMSE	MAE	R^2^	Best Fitness	RMSE	MAE	R^2^	
1	11	30	8	3	10	15	45	167.5	4.970	3.664	0.902	144.080	5.940	4.439	0.866	0.884
2	7	50	8	3	10	13	37	173.9	4.749	3.371	0.910	165.180	5.054	3.444	0.902	0.906
3	9	100	8	3	10	15	37	164.0	5.098	3.839	0.897	151.070	5.619	3.856	0.879	0.888
4	6	150	8	3	10	14	33	155.5	5.430	3.858	0.883	146.890	5.807	4.160	0.872	0.878
**5**	**6**	**200**	**8**	**3**	**10**	**13**	**37**	**180.5**	**4.538**	**3.216**	**0.919**	**167.970**	**4.953**	**3.348**	**0.906**	**0.912**
6	6	200	9	3	10	12	39	172.6	4.793	3.532	0.909	149.420	5.692	4.079	0.878	0.894
7	9	200	10	3	10	18	44	161.9	5.175	3.866	0.894	125.360	6.976	4.735	0.822	0.858
8	7	200	11	3	10	18	46	163.9	5.100	3.366	0.897	144.350	5.927	4.055	0.869	0.883
9	9	200	12	3	10	18	50	163.5	5.114	3.777	0.896	146.180	5.840	4.158	0.870	0.883
10	7	200	8	4	10	21	55	171.5	4.830	3.456	0.907	147.920	5.760	3.845	0.875	0.891
11	9	200	8	5	10	22	64	191.3	4.226	3.054	0.929	149.470	5.689	3.960	0.877	0.903

## Data Availability

The data used in this research has been properly cited and reported in the main text.
